# Probabilistic Ecological Risk Assessment of OCPs, PCBs, and DLCs in the Haihe River, China

**DOI:** 10.1100/tsw.2010.126

**Published:** 2010-07-06

**Authors:** Bin Wang, Gang Yu, Jun Huang, Tai Wang, Hongying Hu

**Affiliations:** ^1^ Department of Environmental Science and Engineering, POPs Research Centre, Tsinghua University, Beijing, China; ^2^ Institute for Sustainability and Peace, United Nations University, Shibuya, Tokyo, Japan

**Keywords:** ecological risk assessment, organochlorine pesticides, persistent organic pollutants, DDTs, HCHs, PCBs, DLCs, the Haihe River, joint probability curve, Monte Carlo–based HQ distribution

## Abstract

The Haihe River is the most seriously polluted river among the seven largest rivers in China. Dichloro-diphenyl-trichloroethanes (DDTs), hexachlorocyclohexanes (HCHs), and PCBs (noncoplanar polychlorinated biphenyls) in the Haihe River, Tianjin were determined using a gas chromatograph – electron capture detector (GC-ECD). Dioxin-like compounds (DLCs) were determined using Chemically Activated LUciferase gene eXpression (CALUX) bioassay. HCH and DDT levels were, respectively, 0.06–6.07 μg/L and ND (not detected) to 1.21 μg/L; PCB levels ranged from 0.12 to 5.29 μg/L; and the total DLCs in sediment were 4.78–343 pg TEQ (toxic equivalency)/g. Aquatic ecological risk assessment was performed using the joint probability curve method and the Monte Carlo-based HQ (hazard quotient) distribution method. The combined risks of similar chemicals and the total risk of dissimilar categories of chemicals were assessed based on the principles of joint toxicity. Due to the adjacent industrial activities, the risk levels of PCBs, DDTs, and HCHs were relatively high. The risk order was as follows: PCBs > DDTs ≈ HCHs > DLCs. The risk of HCHs approximated that of DDTs, which is different from the fact that risk of HCHs is usually much lower in the other Chinese rivers. The total risk caused by these pollutants was very high. Due to their high persistence and potential source from land, the high risks of such pollutants are likely to last for a long period of time.

## INTRODUCTION

China, as one of the countries with the largest agricultural production system, was once a major producer and consumer of organochlorine pesticides (OCPs)[1]. From the 1950s to 1980s, the production of dichloro-diphenyl-trichloroethanes (DDTs) and hexachlorocyclohexanes (HCHs) in China was 0.4 and 4.9 million tons, accounting for 20 and 33% of the total global production, respectively[2]. Polychlorinated biphenyls (PCBs) are another important category of legacy persistent organic pollutants (POPs), whose production in China from 1965 to 1974 was about 10,000 tons[3]. The unintentionally produced legacy POPs, polychlorinated dibenzo-p-dioxins and furans (PCDD/Fs), have been poorly characterized in the environmental matrices in China due to limited instrumentation and trained personnel[4]. The annual dioxin release in China was estimated to be about 10 kg TEQ (toxic equivalents)[5]. Recently, Chemically Activated LUciferase gene eXpression (CALUX) bioassay has been used to determine the PCDD/F pollution in China[6,7]. Compared with the high-resolution gas chromatography/high-resolution mass spectrometry (HRGC/HRMS) method, CALUX is a faster and cheaper method that can provide a comprehensive measure of the aryl hydrocarbon receptor activity and potential hazard[8].

The Haihe River, the largest water body in North China, is 1,329 km long from the longest tributary. It is formed by more than 300 tributaries and converges near Tianjin City to form the 70-km trunk of the Haihe River, which then flows into Bohai Bay. The average annual water flow of the Haihe River is $2.1 \times 10^{10}$ $m^3$. The Haihe River is the most badly polluted river among the seven largest rivers in China[9]. As an important chemical industry city in China, Tianjin has produced huge amounts of OCPs, such as technical HCH, lindane, DDT, chlorobenzene, as well as pentachlorophenol (PCP) and its sodium salt (PCP-Na) for a long period of time[10,11]. PCBs have also been used in Tianjin to produce paints. Chlorinated chemicals, such as PCP and PCP-Na, have been identified as an important source of dioxin release[12,13,14]. The local flourishing chloralkli industry may also unintentionally produce dioxin-like compounds (DLCs)[15].

Although the Haihe River may have been badly polluted by such pollutants, their potential ecological risks have not been well addressed. The conventional method of ecological risk assessment (ERA) is a deterministic approach, such as the hazard quotient (HQ) method, which is suitable for preliminary screening–stage risk assessment. However, it cannot account for the spatial and temporal variability of exposure levels as well as the probability of ecological effects. As an alternative, probabilistic risk assessment, such as the joint probability curve (JPC) method and the Monte Carlo–based HQ distribution method, can provide a quantitative description of the distribution and possibility of ecological risks posed by pollutants in the environment[16,17]. The aquatic ecosystem is usually exposed to mixtures of similar or dissimilar chemicals. The risk caused from the exposures to multiple chemicals should also be cautioned.

This study aims to use probabilistic options to refine the aquatic ERA of various POPs (including OCPs, PCBs, and DLCs) in the Haihe River, China. The risks to aquatic species were assessed using information on their experimentally determined concentration data and reported toxicity values. The combined risks of similar chemicals and the total risk of dissimilar categories of chemicals were assessed based on the principles of joint toxicity. This study can contribute to the risk management of such pollutants.

## MATERIALS AND METHODS

### Sampling and Analysis of the POPs

#### *Sampling*

The sampling sites (Fig. 1) spread from 117°11.00’E to 117°48.50’E, and from 38°58.10’N to 39°07.83’N. Water samples were taken twice, in August 2004 and March 2005, for OCP and PCB analysis. Sediment samples were also taken twice, in March 2005 and November 2006, for DLC analysis. The mass fraction of organic carbon contents ($f_{oc}$) of the sediment samples were determined according to the national standard method of China[18].

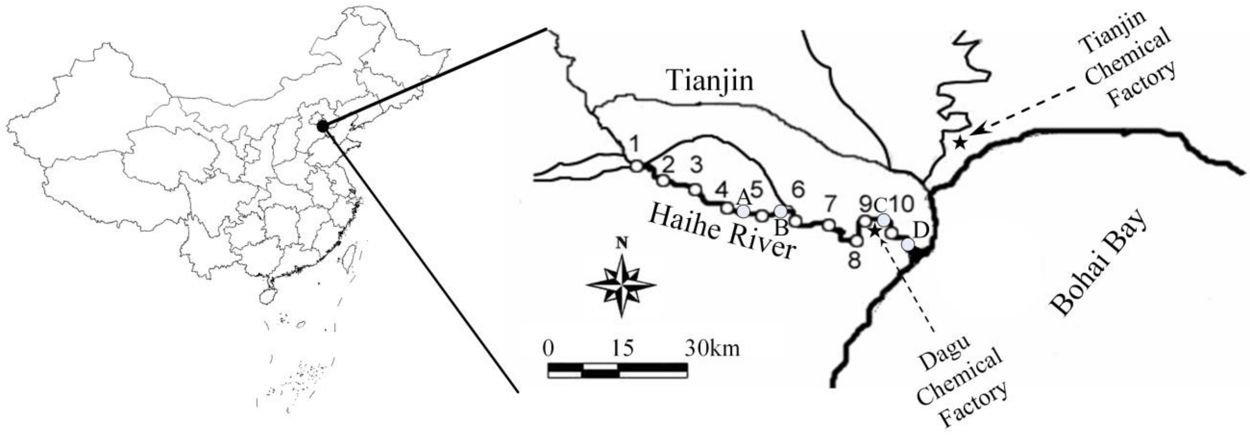

**FIGURE 1.** Sampling sites in the Haihe River. Water and sediment samples were taken from Sites 1–10. Four additional water samples were taken from sites A, B, C, and D in March 2005.

## OCP and PCB Analysis[19]

OCP and PCB analysis was conducted following USEPA method 8081B and USEPA method 8082A, respectively. Water samples were filtered through a glass fiber filter (Millipore, 47-mm diameter, 0.7-µm nominal cutoff). Decabromobiphenyl (PCB209, 0.2 µg) was added to water samples as an internal standard. Then, water samples were passed through the Supelclean ENVI-18 solid-phase extraction (SPE) cartridges at a flow rate of 5 mL/min. Following extraction, the cartridges were freeze dried and eluted with 15 mL of ethyl acetate. The effluents were concentrated to 1.0 mL for the gas chromatograph – electron capture detector (GC-ECD) (Agilent 6890; Agilent Technologies, USA) analysis. The statistical summary of their concentrations are listed in Table 1. If the concentrations were below the limit of detection (LOD), namely ND (not detected), random values were selected at the interval[0, LOD] to substitute for ND, assuming a uniform distribution.

## DLC Analysis[6]

DLC analysis was conducted following USEPA method 4435. The sediment samples were freeze dried, milled, and passed through a 60-mesh sieve; 3.50 g of sediment samples were extracted with methanol/toluene (1:4, v/v), cleaned up by a 25-mL celite column, and a 25-mL sulfuric acid silica gel column, and then separated on a 5-mL disposable X-CARB (Xenobiotic Detection Systems, USA) column into PCDD/Fs fraction and DL-PCBs fraction. H1L6.1c2 cells were exposed to the fractions in 96-well plates. RLU (relative light unit) was then measured by the microplate luminometer (Berthold Centro LB 960, Germany). The calibration curve was used to convert the measured RLU values into CALUX TEQ values expressed in pg TEQ/g dw (dry weight) sediment. HRGC (Agilent 6890N)/HRMS (JEOL JMS-800D, Japan) analysis was also conducted to make validation. Ten 2,3,7,8-substitute PCDFs, seven 2,3,7,8-substitute PCDDs, and 12 DL-PCBs were determined. A good correlation between CALUX results and HRGC/HRMS results was observed. The water concentrations ($C_{water}$) of DLCs were estimated from CALUX TEQs in sediment ($C_{sed}$) based on the equilibrium partitioning method[20]:

$$C_{water}\ (\text{pg TEQ/L}) = C_{sed}\ (\text{pg TEQ/kg})/K_p = C_{sed}\ (\text{pg TEQ/kg}) \cdot f_{oc}/K_{oc} \qquad (1)$$

where $K_p$ is the partition coefficient between the solid and the aqueous phase; L/kg; $K_{oc}$ is the partition coefficient normalized by organic carbon content.

**TABLE 1**
**Statistical Summary of Species Toxicity Data of the Studied POPs and Their Concentrations in the Haihe River**

| | Compound | Statistical Summary | | | | |
|---|---|---|---|---|---|---|
| | | n | Min | Max | Geometric Mean | Geometric SD |
| Toxicity data (μg/L) | p,p'-DDT | 207 | 0.10 | 1,634,840 | 96.95 | 19.66 |
| | p,p'-DDE | 10 | 1.55 | 123,173 | 124.61 | 32.85 |
| | p,p'-DDD | 25 | 0.29 | 22,200 | 46.59 | 17.19 |
| | α-HCH | 18 | 120 | 24,000 | 1938.30 | 5.38 |
| | γ-HCH[a] | 145 | 2.00 | 3,926,205 | 278.44 | 14.73 |
| | δ-HCH | 39 | 6.90 | 100,000 | 683.05 | 13.34 |
| | PCBs | 37 | 0.53 | 38,725 | 112.80 | 18.34 |
| | 2,3,7,8-TCDD | 13 | 0.0017 | 30 | 0.68 | 20.99 |
| Concentration (μg/L) | p,p'-DDT | 24 | ND | 1.05 | 0.0052 | 80.61 |
| | p,p'-DDE | 24 | ND | 0.16 | $2.56 \times 10^{-4}$ | 77.60 |
| | p,p'-DDD | 24 | ND | 0.053 | $1.49 \times 10^{-4}$ | 91.38 |
| | DDTs[b] | 24 | ND | 1.16 | 0.020 | 38.97 |
| | α-HCH | 24 | 0.0079 | 2.72 | 0.10 | 4.07 |
| | γ-HCH[a] | 24 | 0.043 | 2.13 | 0.22 | 2.68 |
| | δ-HCH | 24 | 0.0077 | 1.23 | 0.11 | 3.38 |
| | HCHs[c] | 24 | 0.050 | 3.02 | 0.29 | 2.80 |
| | PCBs | 24 | 0.12 | 5.29 | 0.59 | 2.70 |
| | DLCs[d] | 20 | 4.78 | 343.25 | 35.29 | 3.88 |

[a] γ-HCH and β-HCH were merged into γ-HCH to perform ERA due to the lack of toxicity data of β-HCH.

[b] DDTs value is the total equivalent concentration of DDTs to p,p'-DDT.

[c] HCHs value is the total equivalent concentration of HCHs to γ-HCH.

[d] The unit is pg TEQ/g.

## Ecological Risk Assessment of the POPs

### *Toxicity Data*

Toxicity data (half lethal concentration, $LC_{50}$) of DDTs, HCHs, PCBs, and 2,3,7,8-PCDD for aquatic species in various trophic levels were mainly extracted from Aquatic Information Retrieval of the USEPA (AQUIRE, http://www.epa.gov/ecotox). Species sensitivity distributions (SSDs) were constructed by developing cumulative probability distributions of logarithmically transformed $LC_{50}$[21]. For each species, if more than one toxicity datum was available, the average was taken to develop SSDs. The species toxicity data are summarized in Table 1.

### JPC Method

The Kolmogorov-Smirnov test showed that the lognormal model was generally applicable for fitting the spatial and temporal distribution of the concentrations of these pollutants in the Haihe River. Therefore, the lognormal model was used to fit the exposure concentrations to generate the exposure concentration distributions (ECDs). A JPC was generated from aquatic ECD and SSD[22]. The overall risk was usually illustrated qualitatively by the adjacence degree of the curve to axes. In this study, the overall risk probability (ORP) was introduced to characterize the overall ecological risk quantitatively:

$$ORP = \int_0^1 EPr(x)\,dx \tag{2}$$

where EPr(x) is the exceedance probability for x proportion of species affected ($0 \leq x \leq 1$). ORP could also be calculated as the area under the JPC. Because sufficient chronic toxicity data, such as no observed effect concentrations (NOECs), were unavailable, SSDs were developed based on a set of "surrogate NOECs" generated by dividing acute toxicity values ($LC_{50}$) by a set of ACRs (acute to chronic ratio) (1, 5, 25, 125, and 1000). A series of JPCs and corresponding ORPs was achieved[22].

### Monte Carlo–Based HQ Distribution Method

HQ was defined as follows:

$$HQ = EEC / LC_{50} \tag{3}$$

where EEC is environmental exposure concentration. The HQ distribution was generated from ECD and $LC_{50}$-based SSD after 20,000-times Monte Carlo simulation using Matlab v7.0. Risk was then expressed as the probability of exceeding certain HQ criteria (1, 1/5, 1/25, 1/125, 1/1000) corresponding to a series of ACRs mentioned above[22].

## RESULTS AND DISCUSSION

### Pollution Status of the POPs

In the Haihe River, HCH levels (including α-HCH, β-HCH, γ-HCH, and δ-HCH) were 0.30–1.07 μg/L in August 2004 and 0.06–6.07 μg/L in March 2005. DDT levels (including p,p'-DDT, p,p'-DDD, and p,p'-DDE) were ND to 0.15 μg/L in August 2004 and 0.04–1.21 μg/L in March 2005. HCH levels were generally higher than DDT levels. Total PCB levels ranged from 0.31 to 3.12 μg/L in August 2004 and from 0.12 to 5.29 μg/L in March 2005. The DLC levels (including PCDD/Fs and DL-PCBs) in sediment were 4.78–343 and 5.44–324 pg TEQ/g in March 2005 and November 2006, respectively. PCDD/Fs were the main contributor to the total TEQs, which contributed more than 97% of the total TEQs. Compared with the other rivers in China and worldwide, the levels of such POPs were realtively high in the Haihe River. The highest levels occurred at its downstream area (Sites 9 and 10), which was near the chemical industrial area. The Dagu Chemical factory, located near Sites 9 and 10, was the only legal HCH and lindane producer in China after 1983, and it produced HCHs or lindane until 1999. The Tianjin Chemical factory, as one of the two legal DDT producers in China, produced DDTs until May 2009. PCBs had been used to produce paint at the Tianjin Paint factory, the largest paint factory in China. PCBs can also be released into the water body from the painted ships in the Haihe River and the adjacent Bohai Bay. The PCPs and PCP-Na had been manufactured for a long period of time at the Dagu Chemical factory. It is sure that these industrial sources contributed a lot to the heavy pollution of these POPs.

## Ecological Risk of OCPs

### *Ecological Risk of Single OCPs*

The results of the Monte Carlo–based HQ distribution method are shown in Table 2. From the geometric mean of HQ, it can be seen that the risk of γ-HCH ranked first among the OCPs in the Haihe River. The risk order was γ-HCH > δ-HCH > p,p'-DDT > α-HCH > p,p'-DDD > p,p'-DDE. However, according to the probabilities of exceeding a HQ criterion of 1/25, the risk order was p,p'-DDT > γ-HCH > p,p'-DDD > p,p'-DDE > δ-HCH > α-HCH. The risk order seemed to be different based on different evaluation indicators. The reason might be the different meanings between the geometric mean of HQ and risk probabilities of exceeding a HQ criterion of 1/25. The former is the statistical value of the results of Monte Carlo simulation, regardless of whether HQ is higher or lower than HQ criteria. The latter reflects the part of HQs that may cause potential risk. Risk probability based on such a specific criterion is more risk-meaningful. The ecological risk probabilities also differ a lot based on different HQ criteria. Too loose an ecological protection level (HQ = 1) may cause negligence of risk, and no measure will be taken to reduce the existing risk. On the contrary, risk may be overestimated at a too rigorous ecological protection level (HQ = 1/1000). In order to minimize the risk to a too rigorous criterion, massive manpower, material, and financial resources may be wasted on overprotective measures. Although chronic toxicity data are preferable in ERA, a HQ of 1/25 is a suitable approximate ecological protection level when chronic toxicity data of species are scarce and $LC_{50}$s are used to develop SSD.

**TABLE 2**
**The Results from the Monte Carlo–Based HQ Distribution Method**

| Compound | Geometric Mean of HQ | Probability of Exceeding Preselected HQ Criteria | | | | |
|---|---|---|---|---|---|---|
| | | 1 | 1/5 | 1/25 | 1/125 | 1/1000 |
| α-HCH | $5.43 \times 10^{-5}$ | $3.75 \times 10^{-6}$ | $9.02 \times 10^{-5}$ | $1.30 \times 10^{-3}$ | $1.14 \times 10^{-2}$ | $9.20 \times 10^{-2}$ |
| γ-HCH | $7.76 \times 10^{-4}$ | $6.60 \times 10^{-3}$ | $2.74 \times 10^{-2}$ | $8.62 \times 10^{-2}$ | $2.10 \times 10^{-1}$ | $4.65 \times 10^{-1}$ |
| δ-HCH | $1.67 \times 10^{-4}$ | $1.16 \times 10^{-3}$ | $6.53 \times 10^{-3}$ | $2.75 \times 10^{-2}$ | $8.76 \times 10^{-2}$ | $2.65 \times 10^{-1}$ |
| p,p'-DDT | $5.67 \times 10^{-5}$ | $4.28 \times 10^{-2}$ | $7.55 \times 10^{-2}$ | $1.24 \times 10^{-1}$ | $1.92 \times 10^{-1}$ | $3.07 \times 10^{-1}$ |
| p,p'-DDE | $1.79 \times 10^{-6}$ | $1.07 \times 10^{-3}$ | $2.16 \times 10^{-2}$ | $4.07 \times 10^{-2}$ | $7.18 \times 10^{-2}$ | $1.36 \times 10^{-1}$ |
| p,p'-DDD | $3.07 \times 10^{-6}$ | $9.84 \times 10^{-3}$ | $2.08 \times 10^{-2}$ | $4.08 \times 10^{-2}$ | $7.42 \times 10^{-2}$ | $1.44 \times 10^{-1}$ |
| HCHs | $1.00 \times 10^{-3}$ | $7.99 \times 10^{-3}$ | $3.25 \times 10^{-2}$ | $9.99 \times 10^{-2}$ | $2.36 \times 10^{-1}$ | $5.04 \times 10^{-1}$ |
| DDTs | $2.08 \times 10^{-4}$ | $3.46 \times 10^{-2}$ | $7.04 \times 10^{-2}$ | $1.30 \times 10^{-1}$ | $2.17 \times 10^{-1}$ | $3.68 \times 10^{-1}$ |
| PCBs | $5.37 \times 10^{-3}$ | $4.44 \times 10^{-2}$ | $1.19 \times 10^{-1}$ | $2.56 \times 10^{-1}$ | $4.47 \times 10^{-1}$ | $7.06 \times 10^{-1}$ |
| DLCs | $1.32 \times 10^{-5}$ | $3.56 \times 10^{-4}$ | $1.87 \times 10^{-3}$ | $7.86 \times 10^{-3}$ | $2.68 \times 10^{-2}$ | $9.62 \times 10^{-2}$ |
| Total | | $8.51 \times 10^{-2}$ | $2.09 \times 10^{-1}$ | $4.21 \times 10^{-1}$ | $6.78 \times 10^{-1}$ | $9.17 \times 10^{-1}$ |

The ORPs at various ACRs in the JPC method (Table 3) approximated the probabilities of exceeding the corresponding HQ criteria (Table 2). According to the ORPs at an ACR of 25, the risks of p,p’-DDT and γ-HCH were the highest in the Haihe River, while the risk of α-HCH was the lowest. ORPs calculated at the ACR of 5 and 125 could be respectively considered as the lower limit and higher limit of ORPs calculated at the ACR of 25. The ORPs would be underestimated if $LC_{50}$ were directly used to develop the JPC (ACR = 1) and overestimated if ACR was set as 1000.

**TABLE 3**
**ORPs Calculated from JPCs**

| Compound | ORPs at Various ACRs | | | | |
|---|---|---|---|---|---|
| | 1 | 5 | 25 | 125 | 1000 |
| α-HCH | $3.55 \times 10^{-6}$ | $8.63 \times 10^{-5}$ | $1.26 \times 10^{-3}$ | $1.11 \times 10^{-2}$ | $9.04 \times 10^{-2}$ |
| γ-HCH | $6.41 \times 10^{-3}$ | $2.70 \times 10^{-2}$ | $8.61 \times 10^{-2}$ | $2.11 \times 10^{-1}$ | $4.69 \times 10^{-1}$ |
| δ-HCH | $1.20 \times 10^{-3}$ | $6.67 \times 10^{-3}$ | $2.79 \times 10^{-2}$ | $8.85 \times 10^{-2}$ | $2.66 \times 10^{-1}$ |
| p,p'-DDT | $4.35 \times 10^{-2}$ | $7.62 \times 10^{-2}$ | $1.25 \times 10^{-1}$ | $1.92 \times 10^{-1}$ | $3.06 \times 10^{-1}$ |
| p,p'-DDE | $1.02 \times 10^{-2}$ | $2.08 \times 10^{-2}$ | $3.96 \times 10^{-2}$ | $7.04 \times 10^{-2}$ | $1.34 \times 10^{-1}$ |
| p,p'-DDD | $9.74 \times 10^{-3}$ | $2.07 \times 10^{-2}$ | $4.08 \times 10^{-2}$ | $7.42 \times 10^{-2}$ | $1.44 \times 10^{-1}$ |
| HCHs | $8.57 \times 10^{-3}$ | $3.40 \times 10^{-2}$ | $1.03 \times 10^{-1}$ | $2.40 \times 10^{-1}$ | $5.06 \times 10^{-1}$ |
| DDTs | $3.66 \times 10^{-2}$ | $7.34 \times 10^{-2}$ | $1.33 \times 10^{-1}$ | $2.21 \times 10^{-1}$ | $3.71 \times 10^{-1}$ |
| PCBs | $4.40 \times 10^{-2}$ | $1.18 \times 10^{-1}$ | $2.55 \times 10^{-1}$ | $4.46 \times 10^{-1}$ | $7.06 \times 10^{-1}$ |
| DLCs | $3.61 \times 10^{-4}$ | $1.90 \times 10^{-3}$ | $8.02 \times 10^{-3}$ | $2.73 \times 10^{-2}$ | $9.80 \times 10^{-2}$ |
| Total | $8.71 \times 10^{-2}$ | $2.12 \times 10^{-1}$ | $4.25 \times 10^{-1}$ | $6.81 \times 10^{-1}$ | $9.18 \times 10^{-1}$ |

## Combined Risk of DDTs and HCHs

For similar compounds, the joint toxicity mechanism is usually concentration addition[23,24,25,26]. Thus, total equivalent concentration of the DDTs or HCHs was calculated according to the concept of concentration addition:

$$C_{equ,tol} = \sum_{i=1}^{n} C_{equ,i} = \sum_{i=1}^{n} C_i \times \frac{LC_{50ref,geomean}}{LC_{50i,geomean}} \quad (4)$$

where $C_i$ is the aquatic concentration of compound i; $LC_{50i,geomean}$ and $LC_{50ref,geomean}$ are, respectively, the geometric mean of $LC_{50}$ of compound i and the reference compound. In this study, p,p'-DDT was used as the reference compound for DDTs; γ-HCH was used as the reference compound for HCHs. According to the distribution of $C_{equ,tol}$ and SSD of the reference compound, the combined ecological risks of DDTs and HCHs were assessed by the JPC and HQ distribution methods.

Combined HQ distributions of DDTs and HCHs in the Haihe River are shown in Fig. 2A,B. The geometric means of combined HQs of HCHs and DDTs in the Haihe River were, respectively, $1.00 \times 10^{-3}$ and $2.08 \times 10^{-4}$. The risk probabilities of exceeding a HQ criterion of 1/25 were, respectively, $9.99 \times 10^{-2}$ and $1.30 \times 10^{-1}$ (Table 3). JPCs of DDTs and HCHs in the Haihe River are shown in Fig. 3A,B. The stricter the risk criteria (the larger the ACRs), the higher the calculated risk (the more distance between the curve and the coordinate axis). Fig. 3 shows that the risk level of DDTs was higher than that of HCHs when a stricter criterion was applied; while the risk level of DDTs was less than that of HCHs when a looser criterion was applied. This indicates that the risk criterion cannot only influence the absolute values of risk, but also influences the relative risk order of the pollutants when their risks approximate each other. For the other water bodies in China, the ecological risk of DDTs is usually higher than that of HCHs[27], which is mainly because the aquatic toxicity of DDTs is much higher than that of HCHs[21]. However, in the Haihe River, the ecological risk of HCHs was close to or even higher than that of DDTs. The main reason is that the Dagu Chemical factory (Fig. 1) was the only legal HCH and lindane producer in China after 1983, and the HCH release from the factory contributed to its high risk levels in the Haihe River.

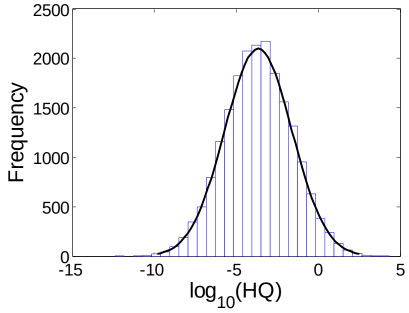

(A) DDTs

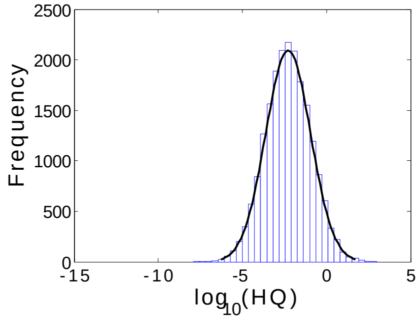

(B) HCHs

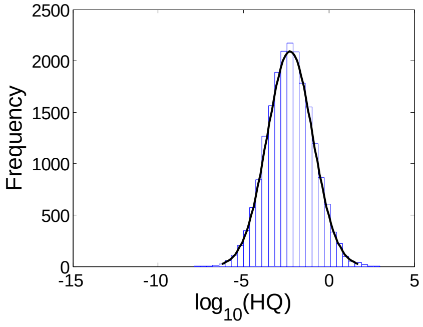

(C) PCBs

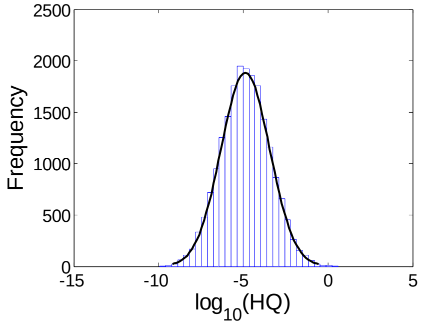

(D) DLCs

**FIGURE** 2. Combined HQ distributions of DDTs, HCHs, PCBs, and DLCs in the Haihe River.

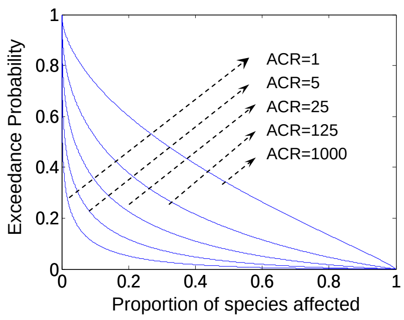

(A) DDTs

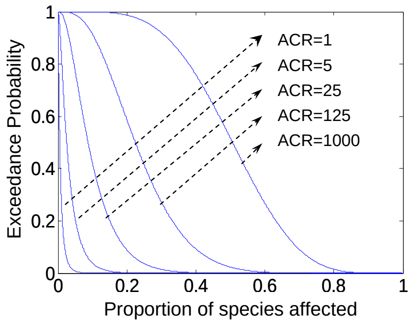

(B) HCHs

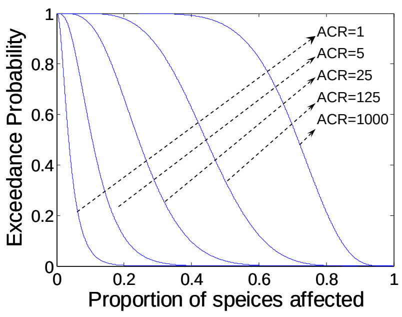

(C) PCBs

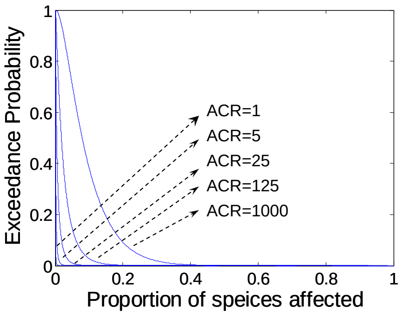

(D) DLCs

**FIGURE 3.** JPCs of DDTs, HCHs, PCBs, and DLCs in the Haihe River.

The combined risks caused by the DDTs or HCHs were significantly higher than the risks of individual chemicals due to their joint action. The joint toxicity mechanism of a mixture composed of similarly acting chemicals is usually concentration addition[23,24,25,26]. In the Haihe River, various similarly acting chemicals coexist, the fractional toxicities of the components remain, and concentration addition effect of the individual components will contribute to the higher combined ecological risk.

## Ecological Risk of PCBs and DLCs

In this study, the risks of individual components of PCBs and DLCs were not assessed due to the lack of enough toxicity data for individual components. The combined risk of PCBs and DLCs are illustrated in Figs. 2 and 3. Table 3 shows that ORPs differed largely for different ACRs. The ORPs calculated at an ACR of 25 were $2.55 \times 10^{-1}$ for PCBs and $8.02 \times 10^{-3}$ for DLCs. The risk of PCBs was higher than that of HCHs and DDTs, while the risk of DLCs was much lower than that of HCHs and DDTs. For PCBs and DLCs, the risk probabilities of exceeding the preselected HQ criteria (Table 2) were also very close to the ORPs at the corresponding ACRs (Table 3). Thus, the same conclusion could be drawn from the HQ distribution method and from the JPC method. They are just the different risk characterization modes with the same essence. However, the JPC method excels the HQ method in its ability to estimate probabilities for different proportions of species affected at various ACRs.

## Total Risk of the POPs

In the Haihe River, the risks of PCBs, HCHs, and DDTs were relatively high, compared with the other water bodies in China[27]. Generally, the risk order was as follows: PCBs > HCHs ≈ DDTs > DLCs. For different categories of similarly acting compounds (DDTs, HCHs, PCBs, and DLCs), it was assumed that the joint toxicity mechanism is independent action[28]. Thus, the independent action principle of joint toxicity was introduced to solve the ERA of dissimilar categories of chemicals:

$$R = 1 - \prod_{i=1}^{n} (1 - R_i) \tag{5}$$

where $R_i$ indicates the ecological risk of a category of similarly acting compounds (DDTs, HCHs, PCBs, or DLCs).

Tables 2 and 3 show that the total risk caused by these POPs was very high. The high risk was certainly related to the past industrial activities near the Haihe River. Fortunately, in Tianjin, DDT production stopped in 2009 and HCH production stopped in 1999. The production of PCP and PCP-Na stopped in 2004. PCBs were also forbidden from being used in the paints. The control of these chlorinated industries can also reduce the unintentional generation of DLCs. However, it was found that DDT and PCB levels in the adjacent marine environment increased in 2006, compared with those in 2005[29]. This indicates that the risk of such legacy POPs still should be cautioned against due to their high levels in the surrounding land-soil matrices[30]. Rainfall and the subsequent surface runoff can bring them from land-soil into the water body. The use of dicofol may also bring a new DDT pollution source[31]. The DDT- and PCB-containing antifouling paints used for boats in the Haihe River can also be an important source of DDT and PCB pollution. In the U.S., the release of PCBs increased by 2.3 million pounds (over 120%) in 2008, due to the removal of PCBs from service and then sent for disposal[32]. In China, there are a large number of PCB-containing facilities sealed at some sites for safekeeping. More attention should be paid to the risk of PCB release to the ambient environment from these stockpile sites or during the PCB disposal process.

Uncertainty still remains even though probabilistic methods are used in ERA. The uncertainty is associated with variability in ecosystem stressors, exposure data, species effect data, risk characterization model, and lack of knowledge[33]. Despite the inherent uncertainties in current risk assessment, the screening for high-risk pollutants would help to decide the allocation of limited manpower, material, and financial resources to priority risk problems.

## CONCLUSIONS

This work determined DDTs, HCHs, PCBs, and DLCs in the Haihe River, and probabilistic approaches were used to analyze their potential ecological risks. The results from Monte Carlo–based HQ distribution and the JPC method were consistent with each other. Due to industrial activities, their pollution levels and risk levels were high. The combined risks caused by DDTs and HCHs were significantly higher than that of individual OCPs due to their joint action. Generally, in the Haihe River, the risk order was as follows: PCBs > HCHs ≈ DDTs > DLCs. The total ecological risk of such POPs was very high. With the prohibition of usage and production of the related chlorinated chemicals, it is anticipated that the risk levels will be reduced in the far future. However, the risk caused by such POPs may still exist for a long period due to their high persistence and potential sources from land. Further monitoring and risk assessment is still merited.

## ACKNOWLEDGMENT

This work was supported by the Chinese National Natural Science Fund for the Distinguished Young Scholars (No. 50625823) and the National Basic Research Program of China (No. 2003CB415007).
